# Leadership Styles in Physical Education: A Longitudinal Study on Students’ Perceptions and Preferences

**DOI:** 10.3390/children12091139

**Published:** 2025-08-28

**Authors:** Adrian Solera-Alfonso, Juan-José Mijarra-Murillo, Romain Marconnot, Miriam García-González, José-Manuel Delfa-de-la-Morena, Pablo Anglada-Monzón, Roberto Ruiz-Barquín

**Affiliations:** 1 Department of Physical Therapy, Occupational Therapy, Physical Medicine and Rehabilitation, Universidad Rey Juan Carlos, Avenida Atenas s/n, 28922 Alcorcon, Spain; adrian.solera@urjc.es (A.S.-A.); juanjo.mijarra@urjc.es (J.-J.M.-M.); miriam.garciag@urjc.es (M.G.-G.); jose.delfa@urjc.es (J.-M.D.-d.-l.-M.); 2 Department Physical Education, Sport and Human Motricity, Universidad Autonoma de Madrid, 28049 Madrid, Spain; pablo.anglada@uam.es; 3 Interfaculty Program, Department of Developmental and Educational Psychology, Universidad Autonoma de Madrid, 28049 Madrid, Spain; roberto.ruiz@uam.es

**Keywords:** leadership, physical education and training, sport, feedback, teaching, pedagogy, longitudinal studies

## Abstract

**Background/Objectives:** Leadership in physical education plays a critical role in the holistic development of students, influencing variables such as satisfaction, group cohesion, and performance. Despite the abundance of cross-sectional studies, there is a paucity of longitudinal evidence exploring the temporal stability of these perceptions in adolescent populations, which limits the current understanding of leadership development in educational settings. This longitudinal study investigates how secondary and high school students perceive and prefer different leadership styles in PE and how these relate to gender, academic level, and sport participation, grounded in the multidimensional leadership model. The analysis is further contextualized by recent research emphasizing adaptive, evidence-based pedagogical approaches in physical education, the influence of competitive environments on leadership expectations, and the role of emotional support in training contexts. **Methods:** Using validated questionnaires (LSS-1 and LSS-2), five dimensions were assessed: Training and Instruction, democratic behavior, autocratic behavior, Social Support, and positive feedback, considering variables such as gender, academic level, and extracurricular sport participation. Data were collected at two time points over a 12-month interval, enabling the identification of temporal patterns in students’ perceptions and preferences. Sampling procedures were clearly defined to enhance transparency and potential replicability, and the choice of a convenience sample from two private schools was justified by accessibility and continuity in longitudinal tracking. Although no a priori power analysis was conducted, the sample size (n = 370) was deemed adequate for the non-parametric analyses employed, with an estimated statistical power ≥ 0.80 for medium effect sizes (Cohen’s d = 0.3–0.5). **Results:** The results revealed a marked preference for leadership styles emphasizing social support and positive feedback, particularly among students engaged in sports. Statistically significant differences (*p* < 0.05) were identified based on gender and academic maturity, with female students favoring democratic behavior and students in the fourth year of compulsory secondary education showing a stronger inclination toward styles prioritizing emotional support. Trends toward statistical significance (*p* < 0.10) were also reported, following precedents in the sport psychology and sport sciences literature, as they provide potentially relevant indications for future research directions. The congruence between perceived and preferred leadership emerged as a key factor in student satisfaction, confirming that adaptive leadership enhances students’ learning experiences and overall well-being. However, this satisfaction was inferred from congruence measures, rather than directly assessed, representing a key methodological limitation. **Conclusions:** This study underscores the importance of physical education teachers tailoring their leadership styles to the individual and group characteristics of their students. The findings align with methodological approaches used in preference hierarchy analyses in sport contexts and support calls for individualized pedagogical strategies observed in sports medicine and training research. By providing longitudinal evidence on leadership perception stability and integrating recent cross-disciplinary findings, the study makes an original contribution to bridging the gap between educational theory and practice. The results address a gap in the literature concerning the temporal stability of leadership perceptions among adolescents, offering a theoretically grounded basis for future research and the design of pedagogical innovations in PE.

## 1. Introduction

The concept of leadership has evolved significantly throughout history, adapting to social, cultural, and technological changes. From early approaches linking leadership to power and authority [1] to more recent theories understanding it as an influence based on interaction and communication [2], leadership has been the subject of extensive analysis. In the field of sports and physical education, this evolution has been particularly notable [2,3,4,5], as these disciplines demand a profound understanding of group dynamics and individual motivation. Recent contributions [6] have emphasized the role of evidence-based pedagogical strategies in PE, highlighting that adaptive leadership styles—those that respond to student needs and contextual demands—are critical for optimizing learning outcomes. In addition, research in competitive sport has shown that incongruence between leadership preferences and perceptions can increase the risk of burnout and negatively affect young athletes’ well-being [7], which reinforces the importance of analyzing congruence in educational contexts such as PE. However, despite the theoretical advances, there is still a limited number of longitudinal investigations in the PE context that examine how students’ leadership perceptions and preferences change over time, which constitutes a relevant gap in the literature and represents the primary focus of this work.

Currently, the study of leadership in physical education holds great significance due to its substantial impact on students’ holistic development and the improvement of educational quality [8]. The importance of effective leadership in this context is well documented in the literature, highlighting the need for educators to develop specific skills to guide both classroom and physical activities [9]. Empirical evidence from competitive sports contexts [10] shows that leadership expectations vary according to the competitive environment, which parallels the differences in preferences observed among students with varying levels of extracurricular sport involvement in this study. This view is consistent with findings from doctoral research analyzing burnout and leadership in young athletes and coaches, where educational and formative implications of leadership styles have been highlighted [11]. This parallel suggests that contextual factors—such as the degree of competitiveness—may act as moderating variables in the relationship between leadership style and student outcomes, a perspective rarely explored in school-based PE research.

This research aims to explore leadership scales from the perspective of students’ perceptions and preferences through a longitudinal study based on data obtained within the framework of a doctoral investigation. The longitudinal design, with two measurement points over a 12-month period, enables an analysis of temporal stability that is seldom addressed in the PE leadership literature and responds directly to the need for studies that go beyond cross-sectional approaches. In line with this objective, the study proposes the following hypotheses: (1) students will demonstrate a significant preference for leadership styles characterized by high levels of social support and positive feedback; (2) differences in leadership perceptions and preferences will emerge according to gender, with female students favoring more democratic styles; (3) academic level will influence preferences, with older students showing a greater inclination towards emotionally supportive leadership; and (4) participation in extracurricular sports will be associated with higher congruence between perceived and preferred leadership styles. The inclusion of the LSS-2 scale is justified by its specificity in measuring students’ preferred leadership behaviors, providing complementary insights to those obtained through the LSS-1 scale, which assesses perceived behaviors. Both scales have been previously validated and are widely used in leadership behavior research [12,13]. Moreover, reporting both perceived and preferred behaviors allows for identifying gaps between expectation and experience, an analytical approach that can inform targeted interventions in PE settings. The methodological framework of this study also draws from preference hierarchy approaches used in performance analysis [14], which enable nuanced interpretations of leadership priorities across demographic subgroups. This dual application allows for a comprehensive comparison between students’ expectations and their actual experiences, a key aspect in understanding leadership effectiveness in educational contexts. This study extends previous research by providing longitudinal evidence of congruence between perceived and preferred leadership styles, an area underrepresented in the current physical education literature. By identifying patterns linked to contextual variables such as gender, academic level, and sport participation, it contributes to a dynamic understanding of the leadership–performance relationship. The emphasis on social support and positive feedback is consistent with findings in adolescent training contexts, where emotional reinforcement has been shown to enhance engagement and progress [15]. In addition, parallels can be drawn with individualized strategies in sports medicine and athlete care [16,17,18], underscoring the value of tailoring leadership approaches to meet diverse participant needs. The findings underscore the necessity for PE teachers to adapt their leadership behaviors to individual student characteristics and offer valuable implications for the design of teacher training programs and instructional strategies. Such adaptations align with recent critiques on the preparation and evaluation of PE teachers [6], reinforcing the practical relevance of this research.

### 1.1. Leadership in History and Its Evolution

The conceptualization of leadership has undergone multiple transformations over time. In antiquity, leadership was primarily associated with military and political power, where leaders made decisions authoritatively and imposed their will on others [19]. This traditional view of leadership as an exercise of power dominated for centuries. However, starting in the 19th century, with the emergence of social and psychological theories, the focus shifted toward understanding how and why certain individuals were more effective in influencing others [3]. As organizations and societies grew more complex, it became evident that leadership was a far more dynamic and interrelated process [4]. Barrow [1] reinforced this shift by defining leadership as a behavioral process of influence that guides groups toward common and specific goals. This perspective aligns with the multidimensional leadership model proposed by Chelladurai & Saleh [20], which defines leadership in physical education and sports contexts as a balance among the leader’s characteristics, group members, the situational context, and the holistic development of individuals.

Recent analyses in sport sciences [6] stress that leadership in educational contexts should not be seen as static or unidirectional but as adaptive and evidence-driven, integrating pedagogical adjustments based on situational feedback. Such perspectives resonate with findings from competitive sport research [10], which reveal that leadership expectations shift according to the competitive intensity and social environment. These insights are especially relevant for the present study, as they justify the inclusion of extracurricular sport participation as a contextual variable likely to influence leadership perceptions and preferences among students.

Recent perspectives conceptualize leadership in sport and PE as a dynamic, context-sensitive process that integrates social and emotional competencies [21,22]. Specifically, Fransen and colleagues [21]. highlight the distinction between task-oriented and motivational leadership roles within teams, showing how these dimensions foster social cohesion and collective efficacy. In PE settings, Beauchamp and Morton [23]. have adapted transformational leadership theory—originally proposed by Bass [24] to educational contexts, demonstrating its relevance for promoting student motivation and engagement. Furthermore, the importance of individualized approaches in leadership mirrors findings in broader physical activity contexts, such as personalized rehabilitation programs for knee osteoarthritis [18] and participant-tailored interventions in sports medicine [16,17], which underscore the value of aligning strategies with individual needs for sustained engagement. This parallel with clinical and training interventions strengthens the argument for adaptive leadership in PE, suggesting that tailoring leadership behaviors to individual characteristics may yield greater adherence, motivation, and learning outcomes.

A systematic review encompassing 2018–2022 found that transformational and autonomy-supportive leadership remain central to contemporary PE programs, with strong implications for student satisfaction and learning outcomes. This is consistent with evidence from adolescent strength training programs [15], where emotional support and constructive feedback significantly enhance adherence and perceived progress. By integrating these diverse strands of evidence, the present research positions itself at the intersection of leadership theory, sport pedagogy, and applied practice, addressing a clear gap in longitudinal analyses of leadership perception stability in adolescent PE contexts.

### 1.2. Leadership in Physical Education: From Sports to Pedagogical Contexts

Sport and physical education (PE) constitute ideal environments for the study of leadership, given their inherent emphasis on interpersonal dynamics, motivation, and performance. In both domains, instructors assume roles that go beyond technical instruction; they shape students’ cognitive, social, and emotional development. This study is based on Chelladurai’s Multidimensional Leadership Model [12,25], which proposes that leadership in these contexts is not only task-oriented but also relational, involving affective support, feedback, and decision-making. By integrating both the task and relational dimensions, this model allows for a more holistic assessment of leadership effectiveness in PE, which is especially relevant when aiming to understand adolescent development over time.

Chelladurai’s Multidimensional Leadership Model is one of the most influential theoretical frameworks in this area. It posits that effective leadership arises from the congruence between three behavior dimensions: required (determined by the environment), preferred (expected by group members), and actual (demonstrated by the leader). Within this model, interaction styles—training and instruction, social support, and positive feedback—and decision-making styles—Democratic and Autocratic—are used to assess leadership effectiveness. Numerous studies have confirmed that when actual behaviors align with student preferences and situational demands, positive outcomes such as increased satisfaction, cohesion, and motivation are observed. Recent methodological applications in sport performance research, such as the Analytic Hierarchy Process [14], provide a useful parallel for understanding how different demographic groups prioritize leadership dimensions, offering a structured approach to interpreting the differential preferences observed in this study across gender and academic levels. This methodological analogy supports the decision to include multiple demographic variables in the analysis, allowing for a richer and more nuanced interpretation of leadership patterns in PE settings.

In PE specifically, students tend to value leadership styles characterized by high levels of emotional support and constructive feedback. These preferences often vary by demographic factors: for instance, female students show a greater inclination toward democratic leadership, while older students appear more receptive to directive styles. Furthermore, participation in structured sports—whether recreational, federated, or competitive—has been shown to influence students’ expectations regarding leadership behavior, reinforcing the need for differentiated pedagogical approaches [23,24,26]. Federated athletes were defined as those registered with an official regional or national sports federation and regularly participating in official competitions. High-performance athletes were enrolled in specialized training programs recognized by sports authorities, with intensified training demands and a focus on elite-level competition. Evidence from competitive sport environments [10] corroborates these distinctions, showing that higher competition levels are associated with stronger preferences for structured and supportive leadership. These distinctions also justify the separation of participants by sports involvement in the present study, as they are likely to reflect different motivational climates and leadership expectations.

Despite the growing body of research in this field, most existing studies employ cross-sectional designs, limiting our understanding of how leadership perceptions and preferences evolve over time. This study addresses this gap by employing a longitudinal approach in the underexplored context of Spanish secondary education. It examines how student perceptions of leadership change across one academic year and how variables such as gender, academic level, and sport participation interact with these dynamics. In doing so, it provides empirical evidence on temporal stability and change in leadership-related perceptions, an aspect scarcely documented in the literature. Such a longitudinal tracking of preference–perception congruence is aligned with calls from recent educational sport science research [6] to monitor leadership effectiveness over extended periods, ensuring that pedagogical strategies remain aligned with student needs. This design also responds to the broader academic demand for methodological approaches capable of detecting subtle but meaningful trends, including those approaching statistical significance, which may inform targeted interventions in future educational practice.

### 1.3. Relevance and Scope of the Study

The relevance of studying leadership within the context of physical education lies in its impact on the development of motor, cognitive, and social skills of students [4,5]. Since physical education is characterized by working on both individual and group performance, the teacher’s leadership plays a key role in the teaching–learning process [4,5]. Moreover, it is essential to consider that individual characteristics of students, such as gender, age, and motor competence level, may influence their leadership preferences and perceptions, highlighting the importance of adapting leadership styles to the needs of each group [27,28]. Recent findings in adolescent training interventions [15] further demonstrate that tailoring pedagogical strategies to individual needs, particularly through emotional reinforcement—enhances engagement and perceived progress, reinforcing the necessity of adaptive leadership in PE. This study expands on these findings by analyzing such dynamics longitudinally, thereby providing evidence not only of the presence of these associations but also of their temporal stability or evolution.

This study is also justified by the need to better understand how the relationship between leaders and followers evolves over time, particularly in a dynamic environment like physical education. Through a longitudinal analysis, it is possible to identify stable patterns of behavior, as well as changes in students’ perceptions and preferences regarding their teachers’ leadership. Such an approach addresses the current shortage of longitudinal data in PE leadership research, a gap that limits the development of evidence-based teacher training programs. Such an approach responds to methodological recommendations in contemporary sport sciences [6], which advocate for the sustained monitoring of teaching effectiveness to ensure alignment with evolving learner needs. Moreover, the competitive context has been shown to influence leadership expectations [10], suggesting that sport participation may be a decisive factor in shaping leadership preferences and perceptions. By integrating this contextual factor into the analysis, the present research acknowledges the multifactorial nature of leadership effectiveness and moves beyond single-variable approaches common in earlier studies. These findings may have practical implications for improving teacher training and, ultimately, optimizing educational outcomes in the field of physical education.

In addition, parallels can be drawn with individualized strategies in sports medicine and rehabilitation, where matching intervention approaches to participant profiles—such as in ACL injury recovery [18], efficacy-focused treatment evaluations [16], and adherence-improving modalities like whole-body cryotherapy [17]—has been shown to increase long-term engagement and satisfaction. These analogies underscore that the same principle applies in PE leadership: alignment between instructional style and student preference is key for sustained motivation and performance. This cross-disciplinary perspective strengthens the argument that PE leadership research can benefit from methodological and conceptual advances developed in other areas of sport and health sciences.

Recent research in sport sciences has underscored the need for adaptive and context-sensitive leadership in physical education. Studies have examined how leadership expectations vary across competitive contexts [10], the impact of emotional reinforcement on adolescent engagement [15], and the methodological advantages of hierarchical preference analysis [14]. Furthermore, broader sport science research on individualized approaches to performance and rehabilitation [16,17,18] supports the importance of aligning instructional strategies with participant needs. By synthesizing these strands of evidence and applying them within a longitudinal design in the Spanish secondary education context, this study provides an original and contextually grounded contribution to the literature on leadership in PE.

In contrast to prior research on leadership in physical education, which has predominantly relied on cross-sectional or short-term designs, the present study provides longitudinal evidence on the stability and evolution of leadership perceptions and preferences over an entire academic year in secondary education. While longitudinal approaches have been applied in other domains of sport sciences, their application to the school PE context—particularly within the Spanish educational system—remains scarce. This focus not only addresses a clear gap in the literature but also offers a differentiated contribution by integrating competitive sport experience, gender, and academic level as interacting contextual variables.

### 1.4. Objectives of the Study

The main objective of this longitudinal study is to analyze leadership within the field of physical education, specifically from the perspectives of students’ preferences and perceptions of their teachers’ leadership styles. The central research question guiding this study is as follows: “How do students’ perceptions and preferences regarding their physical education teachers’ leadership styles vary over time and according to contextual variables such as gender, academic level, and sports participation?”.

This objective addresses a critical gap identified in the PE leadership literature, in which most studies rely on cross-sectional designs and do not assess temporal stability or evolution of leadership perceptions [6]. By focusing on the longitudinal dimension, the study is able to capture both stable tendencies and potential shifts in leadership-related attitudes, offering a more nuanced understanding than cross-sectional approaches. By integrating recent evidence on the influence of competitive environments [10], preference hierarchies across demographics [14], and the role of emotional reinforcement in adolescent training contexts [15], the present work is designed to provide both theoretical and applied insights for educators and policy-makers. Furthermore, the study aims to generate actionable recommendations for teacher training programs, grounded in empirical evidence, to enhance the alignment between instructional strategies and student needs in diverse educational settings.

### 1.5. Research Hypotheses

Based on previous research grounded in Chelladurai’s Multidimensional Leadership Model and the principles of Self-Determination Theory, the following hypotheses were formulated:

**H1.**  *Students will show a greater preference for social support and positive feedback leadership styles, particularly among those involved in extracurricular sports. This expectation is supported by empirical findings indicating that emotional reinforcement and constructive feedback contribute to higher engagement and perceived progress in adolescent PE contexts [15].*

**H2.**  *Female students will express a stronger preference for democratic behavior and social support compared to male students. This prediction aligns with prior literature highlighting gender-related differences in leadership expectations, in which female participants tend to value participative decision-making and relational support more highly [23].*

**H3.**  *The congruence between perceived and preferred leadership styles will be associated with higher levels of student satisfaction. Although satisfaction was not directly measured in this study, this hypothesis is theoretically supported by prior work on individualized pedagogical strategies in both PE and broader sports science interventions [6,16,17]. The underlying assumption is that the alignment between student expectations and teacher behaviors fosters a motivational climate conducive to learning and sustained participation.*

**H4.**  *Leadership preferences and perceptions will remain stable across the two time points measured, indicating consistency over the academic year. This stability hypothesis is methodologically comparable to the preference hierarchy consistency observed in sport performance analysis [14]. Identifying whether such stability exists in the PE context addresses a current gap in longitudinal leadership research and provides valuable insight for designing interventions that either maintain effective practices or adjust ineffective ones.*

## 2. Materials and Methods

### 2.1. Study Design

This study adopted a longitudinal design, which allowed for the analysis of leadership perceptions and preferences at different points in time. The design is descriptive and correlational [29,30], focused on examining the relationship between students’ psychological variables, leadership perceptions and preferences, and academic performance. Specifically, a panel study design was used, whereby the same cohort of students was assessed at two different time points, separated by an interval of 12 months. This methodological approach enabled the examination of intra-individual changes and the evaluation of stability or variability in leadership perceptions and preferences over time.

The decision to employ a longitudinal, rather than cross-sectional, design responds directly to the scarcity of temporal analyses in PE leadership research, allowing the detection of both stable patterns and meaningful changes in student attitudes. This design also facilitates the examination of potential moderating effects of contextual variables such as gender, academic level, and sport participation.

Although no a priori power analysis was conducted, the sample size (n = 370) was estimated to provide ≥0.80 statistical power to detect medium effect sizes (Cohen’s d = 0.3–0.5) in non-parametric tests such as the Mann–Whitney U and Kruskal–Wallis. The choice of non-parametric methods was based on the non-normal distribution of the leadership scale scores, verified through Shapiro–Wilk tests. While the statistical approach is primarily descriptive-comparative, the longitudinal nature of the design enhances interpretive depth by enabling within-subject comparisons over time.

A methodological limitation of this design is the use of only two measurement points, which, while sufficient to capture short-term stability, may overlook more nuanced temporal trajectories. Future research could address this by incorporating additional time points and mixed-methods approaches to better understand the mechanisms underlying observed changes.

### 2.2. Participants

Participants were drawn from two private schools located in urban areas of central Spain. Both institutions serve predominantly middle- to upper-middle-class families, providing a relatively homogeneous socioeconomic profile. This socioeconomic homogeneity should be considered when interpreting results, as it may limit the generalizability of the findings to public-school contexts or populations with more diverse backgrounds. In both schools, students attended two physical education classes per week, each lasting approximately 55 min, in accordance with the regional curriculum. Participation was voluntary and required written informed consent from both students and their legal guardians.

The participants in this study were selected based on specific inclusion criteria to ensure sample homogeneity and the relevance of the obtained data. The inclusion criteria were as follows: (1) students from compulsory secondary education and/or the first year of upper secondary education, (2) students who regularly attended physical education classes (i.e., attended at least 80% of scheduled sessions during the academic term), (3) students who volunteered to participate, and (4) students who signed the informed consent form. The exclusion criteria included any medical condition or injury preventing full participation in PE activities during the measurement periods, as well as incomplete questionnaire responses that would compromise data validity.

No a priori power analysis was conducted. However, with n = 370 at the baseline, the sample size was adequate to detect medium effect sizes (Cohen’s d ≈ 0.3–0.5) with a power ≥ 0.80 in Mann–Whitney U and Kruskal–Wallis tests (α = 0.05). Attrition reduced the available sample to n = 208–221 for some longitudinal analyses, which may have reduced the statistical power for subgroup comparisons. Participant attrition was primarily due to school transfers, prolonged illness, or absence during the second measurement, and its potential impact on the stability of longitudinal estimates should be acknowledged.

### 2.3. Instruments

Two validated questionnaires from the Leadership Scale for Sport [20], adapted into Spanish by [31], were used to collect data and measure students’ leadership preferences (LSS1) and leadership perceptions (LSS2) regarding their physical education teachers (Appendices 1 and 2). Each questionnaire consists of 40 questions representing five different items: (1) training and instruction, (2) democratic behavior, (3) autocratic behavior, (4) social support, and (5) positive feedback. All psychometric scale scores (LSS-1, LSS-2) are reported in arbitrary units (a.u.), consistent with their standard scoring procedure. A questionnaire on sports and sociodemographic data [31] was also used to complete the study variables. This additional instrument gathered information on gender, academic level, the type and frequency of extracurricular sport participation, and competitive involvement (recreational, federated, or high-performance), allowing for subgroup analyses.

The internal consistency (Cronbach’s alpha) coefficients for this sample were as follows: training and instruction (α = 0.82), democratic behavior (α = 0.78), autocratic behavior (α = 0.71), social support (α = 0.84), and positive feedback (α = 0.79), indicating acceptable-to-good reliability across all dimensions. These values are consistent with previous studies using the LSS in PE and sports contexts [12,13], supporting the robustness of the measurement tools in the present sample.

### 2.4. Procedure

Firstly, contact was made with the participating educational centers at the beginning of the academic year, through which the research objectives were explained to the school management teams and the physical education teachers involved. During these meetings, the protocol for administering the questionnaires was presented, clarifying any questions regarding their application and ensuring ethical commitment to the data collection process.

All participating teachers received written instructions outlining the standardized administration procedure, including the order of questionnaires, the exact wording to be used when providing instructions to students, and the time allowed for completion. This was done to minimize interviewer effects and ensure procedural consistency across the two schools. Data collection was carried out during regular PE class time in a quiet environment, with the researcher present to address procedural queries but without influencing responses.

The two measurement points were scheduled 12 months apart, with the first assessment taking place during the first academic term and the second during the same term of the following year, to control for seasonal variations in the school calendar. In one of the schools, minor deviations in timing occurred due to scheduling constraints; this was recorded and considered in the interpretation of results.

Incomplete questionnaires were handled according to a conservative missing-data protocol: if a participant left fewer than 10% of items unanswered within a subscale, the missing responses were replaced using mean substitution for that subscale; otherwise, the participant’s data for that subscale were excluded from analysis. While this approach preserves sample size, it is recognized as suboptimal compared to more advanced imputation techniques such as multiple imputation or maximum likelihood estimation, which are recommended for future research.

### 2.5. Informed Consent

Before starting data collection, informed consent was obtained from the parents or legal guardians of the underage students, as well as from the students and teachers themselves. The study adhered to the ethical principles outlined in Section IV (arts. 33–38) of the Code of Ethics of Psychology of the Official College of Psychologists of Spain (2015), which includes the necessary authorization for participation and the explicit approval from families. Consent forms provided a detailed description of the study’s objectives, procedures, potential risks, and expected benefits, as well as assurances regarding the voluntary nature of participation and the right to withdraw at any time without penalty. Additionally, the research complied with the Organic Law 3/2018 on the Protection of Personal Data, ensuring confidentiality and the exclusive use of the information for research purposes, as well as the ethical and deontological principles of the Declaration of Helsinki (2000). All consent forms were stored securely in locked cabinets, and digital data were anonymized using participant codes before analysis to prevent identification. Ethical approval for the study protocol was obtained from the relevant institutional ethics committee prior to the commencement of data collection.

### 2.6. Administration of Questionnaires

The questionnaires were administered at two points during the academic year. The first point (Time 1) was in October 2018, when the evaluation instruments were applied in physical education classes. Prior to data collection, all teachers responsible for administering the questionnaires participated in a structured training session led by the research team, which included detailed written guidelines, a standardized verbal script, and practice scenarios to ensure uniformity in delivery and reduce interviewer bias. Teachers responsible for administering the questionnaires were trained, as the success of the research depended on the active collaboration of the educators, and teachers were instructed to refrain from influencing student answers in any way.

The second point (Time 2) of data collection varied by educational center. Some schools conducted the second round of questionnaires in April 2019, while others did so in October 2019. This Time 2 allowed for the assessment of changes or stability in students’ leadership perceptions and preferences over time. The variation in timing was due to differences in each school’s internal calendar; these deviations were documented and later considered in the interpretation of results as a potential source of variability. During both phases, a suitable environment was ensured for student participation, guaranteeing the comprehension of the questions and encouraging honest responses. Students completed the questionnaires in quiet classroom conditions during regular PE lesson time, with the researcher present to address clarifying questions about instructions but without providing feedback on item content.

Although slight differences in the implementation timeline between the participating schools may introduce variability, standardized administration procedures and identical instruments were employed to minimize inconsistencies and ensure methodological reliability. This procedural consistency was intended to reduce measurement error and enhance comparability across the two time points.

### 2.7. Data Analysis

To carry out the analysis of the data obtained in this study, SPSS software version 22.0 was used. The analyses applied were carefully selected to address the research objectives, exploring both differences between leadership perceptions and preferences, as well as relationships between key variables. The techniques employed are outlined below:

1. Descriptive analysis: Frequency analyses, measures of central tendency (means), and measures of dispersion (standard deviation) were applied to describe the general profiles of participants in terms of perceived and preferred leadership. These analyses provided an overall view of the responses, highlighting the most common trends in perceptions and preferences. Where relevant, 95% confidence intervals were calculated to provide a range estimate for the observed means.
2. Normality tests: To determine whether the data followed a normal distribution, the Kolmogorov–Smirnov and Shapiro–Wilk tests were applied. Since many variables did not show a normal distribution, non-parametric statistical tests were chosen for subsequent analyses.
3. Mean differences analysis: several non-parametric tests were used to compare mean differences:
  - Mann–Whitney U test: Used to compare two independent samples, such as gender differences.
  - Wilcoxon signed-rank test: Used to compare responses at two different times (pre- and post-test) within the same group of students.
  - Kruskal–Wallis Test: Used to compare multiple independent samples, such as levels of perceived and preferred leadership across different academic levels. For Kruskal–Wallis tests, eta-squared (η^2^) effect sizes were reported to indicate the magnitude of the differences.
4. Instrument reliability: the Cronbach’s alpha coefficient was applied to assess the internal consistency of the questionnaires used.
5. Correlational analysis: Spearman’s correlation coefficients were used to explore the relationships between psychological variables, such as self-efficacy and values, with students’ perceptions and preferences of leadership. These analyses helped identify potential associations between students’ individual characteristics and their preferences for certain leadership styles. Effect sizes for correlations (Spearman’s rho) were interpreted according to conventional thresholds (with 0.10 being small, 0.30 being medium, and 0.50 being large).

Regarding statistical significance, both conventional thresholds (*p* < 0.05) and trends toward significance (*p* < 0.10) were reported. The inclusion of *p* < 0.10 values are justified by three main reasons: (1) they provide valuable preliminary evidence for variables that may warrant further investigation; (2) they indicate potentially important patterns in mean difference and correlation analyses; and (3) this practice has been used in prior relevant studies in sport psychology and sport sciences, including research applying Chelladurai’s Multidimensional Leadership Model [7,11].

Given the descriptive focus of this study, no inferential models (e.g., regression, mediation, or moderation analyses) were conducted. This decision reflects the objective of identifying preliminary trends, rather than establishing predictive or causal relationships. However, future research should incorporate multivariate modeling to explore potential mediating or moderating effects between leadership perceptions, preferences, and contextual variables.

### 2.8. Handling of Missing and Outlier Data

Before proceeding with the statistical analyses, a data-cleaning process was conducted to identify potential outliers and errors in the transcription of responses. Additionally, cases where students omitted certain items in the questionnaires were addressed. Despite the presence of some missing data, it was decided not to exclude participants with incomplete responses to maximize the final sample size. Participants with more than 10% missing data on the LSS items were excluded from analysis (n = 7). For the remaining cases with isolated missing responses (less than 5% of items per participant), missing values within each LSS subscale were replaced by the participant’s mean score for that subscale. This conservative approach was adopted due to the low percentage of missing data (<5%) and to preserve statistical power, particularly important in longitudinal designs where attrition can already reduce sample size.

Although this imputation strategy allowed for the retention of more cases than listwise deletion, it is recognized as suboptimal compared to modern missing-data techniques such as multiple imputation or maximum likelihood estimation, which are recommended for future studies to reduce potential bias and better estimate variability.

Potential univariate outliers were identified using standardized z-scores (>|3.29|) and multivariate outliers with the Mahalanobis distance (*p* < 0.001). Outlier values were examined individually to determine whether they reflected data entry errors or genuine extreme responses; only confirmed errors were corrected, while valid extreme cases were retained in the dataset.

This conservative approach was chosen due to the low percentage of data (<5%) missing values within each LSS subscale were replaced with the participant’s mean score for that subscale. This conservative approach was chosen due to the low percentage of missing data (<5%) and to preserve statistical power. However, more robust methods such as multiple imputation or maximum likelihood estimation are recommended for future studies. Although this conservative imputation was chosen to preserve statistical power, given the sample size, more robust techniques such as multiple imputation or maximum likelihood estimation are recommended for future studies.

This strategy was chosen over listwise deletion to preserve statistical power and representativeness, given the relatively small sample and the longitudinal nature of the design.

The analysis of the data collected at both points (pre- and post-test) provided a comprehensive view of perceived and preferred leadership in physical education, offering a solid foundation for the interpretation and discussion of the results.

## 3. Results

A total of 370 students from compulsory secondary education and first year of upper secondary education stages were included in the study, from two private schools in the Madrid and Castilla-La-Mancha regions (Table 1).

The participants had a mean age (Mage) of 14.48 years (standard deviation [SD] = 3.02), and they were enrolled in secondary education or the first year of the Spanish Baccalaureate. Of these, 48.6% were male (n = 180), and 51.4% were female (n = 190).

Before descriptive analyses, mean difference tests, and correlational analyses were conducted, the Kolmogorov–Smirnov test was applied to the various psychological and values scales administered at Time 1. The results indicated significant deviations from normality across most variables (*p* < 0.05), thus justifying the use of non-parametric procedures for subsequent analyses.

Table 2, Table 3, Table 4, Table 5, Table 6, Table 7 and Table 8 present the results derived from the first questionnaire (LSS-1), which assessed students’ preferred leadership behaviors (in a.u.). These analyses include descriptive statistics, reliability indices, and comparisons based on sex, age, sport modality, and level of sport participation.

The results indicate that the variables analyzed in the students do not follow a normal distribution (*p* < 0.05). Regarding students’ leadership preferences concerning their physical education teacher, as assessed with the LSS-1 questionnaire, the following scores were obtained: training and instruction (KS = 0.093, *p* < 0.001), democratic behavior (KS = 0.072, *p* < 0.01), autocratic behavior (KS = 0.100, *p* < 0.001), social support (KS = 0.069, *p* < 0.05), and positive feedback (KS = 0.091, *p* < 0.001).

On the other hand, in the LSS-2 questionnaire, which measures students’ perceptions of their teacher’s leadership behaviors (in a.u.), the following scores were found: training and instruction (KS = 0.103, *p* < 0.001), democratic behavior (KS = 0.068, *p* < 0.05), autocratic behavior (KS = 0.078, *p* < 0.01), social support (KS = 0.072, *p* < 0.05), and positive feedback (KS = 0.109, *p* < 0.001).

The results of the longitudinal study reveal important trends in the perception and preferences of leadership among physical education students, showing congruences in several styles, especially in social support and positive feedback.

### 3.1. Leadership Perceptions and Preferences

It was observed that, in general, students showed a clear preference (LSS-1) for leadership styles that emphasize social support and positive feedback at Time 1 (Table 2).

It was also observed that students perceived (LSS-2) their teachers as having leadership styles focused on social support and positive feedback at Time 1 (Table 3).

When the mean differences in preferences (LSS-1) and perceptions (LSS-2) of students based on Times 1 and 2 (Table 4 and Table 5) were analyzed using the Wilcoxon signed-rank test, no statistically significant results were observed.

### 3.2. Gender Differences

The results showed statistically significant differences based on students’ gender at Time 1. Although both sexes showed a greater preference for leadership styles such as training and instruction and social support, the most notable differences between boys and girls were found in the democratic behavior style, for which girls expressed a higher preference than boys (Table 6).

Regarding leadership perception at Time 2, it was observed that girls perceive a greater presence of democratic behavior, positive feedback, and training and instruction compared to boys. Although some comparisons yielded *p*-values between 0.05 and 0.10, these were interpreted as statistical tendencies, rather than robust effects. The statistical differences in these styles are more significant at Time 2 (LSS-2) than at Time 1 (LSS-1), highlighting a change in leadership perception over time (Table 7).

### 3.3. Differences by Academic Year

When focusing on the differences by academic year, a significant variation in leadership perception was observed based on the year of study. Two groupings can be identified: on the one hand, for first, second, and third-year students of secondary education, the mean scores showed a continuous decline, and on the other hand, fourth-year secondary education and first-year secondary education students’ mean scores increased.

It was also observed that students in higher years (fourth-year and first-year high school) showed a greater inclination towards leadership styles focused on social support, positive feedback, and autocratic behavior compared to students in lower years. However, higher-year students preferred leadership styles more oriented toward training and instruction, as well as social support. With a focus on statistically significant differences, it was found that there were greater statistical differences between years in the styles of training and instruction, social support, and autocratic behavior (Table 8).

With a focus on leadership perception, it can be observed that the highest mean scores were obtained in the second year, rather than in the first year. Additionally, two course groupings still exist: first-, second-, and third-year students, with decreasing mean scores, and fourth-year and first-year high school students, for whom the mean scores increased with three styles, except in autocratic behavior and social support, for which the mean scores decreased (Table 9). Statistically significant differences were more pronounced in LSS-2 than in LSS-1.

When the variables of academic year and gender were compared, no significant differences were observed in the total sample for both LSS-1 (Table 10 and Table 11) and LSS-2 (Table 12 and Table 13).

### 3.4. Differences by Participation in Physical Activity and Sports, and Level of Practice

The analysis of data based on the LSS-1 questionnaire revealed interesting differences between students who engage in extracurricular sports and those who do not. In general, students who participate in extracurricular sports scored higher on all leadership factors, except for the training and instruction factor, where non-practicing students achieved slightly higher scores. The most significant difference was observed in the social support factor (*p* < 0.05), indicating that athletes value leadership that emphasizes emotional and social support. While the factors of democratic behavior, autocratic behavior, and positive feedback also showed higher scores in athletes, these differences were not statistically significant. Regarding the level of sport participation (recreational, school-based, federated, and elite performance), the results from the LSS-1 questionnaire indicated that students with higher levels of athletic performance tended to score higher on most leadership factors. Elite athletes particularly stood out in the training and instruction and positive feedback factors (*p* < 0.05). The only factor for which no significant differences were found between levels of participation was autocratic behavior (Table 14 and Table 15).

The analysis using the LSS-2 questionnaire showed trends similar to those observed with LSS-1, although with some variations. Students who engage in extracurricular sports scored higher on almost all leadership factors compared to non-practitioners, but none of these differences reached statistical significance. Regarding the level of sport participation, the results indicated that higher levels of performance were associated with higher scores in factors such as training and instruction, democratic behavior (*p* < 0.05), and positive feedback (*p* < 0.05). Although high-performance students excelled again in these factors, no significant differences were found in the factors of autocratic behavior or social support across different levels of participation (Table 16 and Table 17).

### 3.5. Consistency Between Perceived and Preferred Leadership (Table 18, Table 19, Table 20, Table 21 and Table 22)

The study results reflect significant variations in leadership perception over time (Time 1 and Time 2), highlighting differences between perception and preference evaluations (LSS-1 and LSS-2). In the analysis of the LSS-2 minus LSS-1 differential descriptives (Table 18), relevant changes in leadership perception were identified. Specifically, notable differences were observed based on gender, with boys showing a greater differential compared to girls in the styles of autocratic behavior, social support, and positive feedback, suggesting a gender effect on the evaluation and perception of leadership styles.

Regarding specific leadership factors, the congruencies between perceived leadership styles showed variable results. In the training and instruction factor, 70.1% of participants reported high congruence, while in the democratic behavior factor, this percentage increased to 71.1%, 77.9% in autocratic behavior, 75.5% in social support, and 73.5% in positive feedback

A particularly relevant finding concerns the congruence between perceived and preferred leadership styles. Only 36.3% of participants exhibited full congruence across all five dimensions in both time points (Table 19, Table 20, Table 21 and Table 22). This result highlights the diversity in the perception of leadership styles among participants, emphasizing the need to consider individual and sociodemographic differences, such as gender, in future research on leadership and its impact in pedagogical settings.

**Table 18:** Descriptive statistics of the differences LSS-2 minus LSS-1 at Time 1.

	n	M	SD	Minimum	Maximum
LSS-2/LSS-1 Training and Instruction T1	204	0.2794	6.89008	−25.00	20.00
LSS-2/LSS-1 Democratic Behavior T1		0.1029	5.91518	−16.00	17.00
LSS-2/LSS-1 Autocratic Behavior T1		0.4069	3.72532	−11.00	15.00
LSS-2/LSS-1 Social Support T1		−0.2451	5.19081	−15.00	15.00
LSS-2/LSS-1 Positive Feedback T1		−0.0539	4.09397	−14.00	10.00

M: mean; SD: standard deviation.

**Table 19:** Descriptive statistics of the differences LSS-2 minus LSS-1 at Time 2.

	n	M	SD	Minimum	Maximum
LSS-2/LSS-1 Training and Instruction T2	111	0.6577	6.52477	−26.00	20.00
LSS-2/LSS-1 Democratic Behavior T2		−0.5946	5.58957	−14.00	20.00
LSS-2/LSS-1 Autocratic Behavior T2		0.1441	3.50544	−13.00	11.00
LSS-2/LSS-1 Social Support T2		0.3694	4.51850	−15.00	14.00
LSS-2/LSS-1 Positive Feedback T2		−0.2162	3.82909	−14.00	9.00

M: mean; SD: standard deviation.

**Table 20:** Descriptive statistics and analysis of mean differences considering the LSS-1 and LSS-2 scales at Time 1 based on the gender variable (total n = 204).

	Gender	n	M	SD	Average Range	Total of Ranks	Mann–Whitney U	Z	Sig.
LSS-2/LSS-1 Training and Instruction T1	Male	101	0.9901	6.84470	107.14	10,821.00	4733.000	−1.113	0.266
	Female	103	−0.4175	6.89633	97.95	10,089.00			
LSS-2/LSS-1 Democratic Behavior T1	Male	101	1.1485	5.85557	112.27	11,339.00	4215.000	−2.345	0.019 *
	Female	103	−0.9223	5.82033	92.92	9571.00			
LSS-2/LSS-1 Autocratic Behavior T1	Male	101	0.3168	3.55789	102.50	10,352.00	5201.000	−0.001	0.999
	Female	103	−4951	3.89790	102.50	10,558.00			
LSS-2/LSS-1 Social Support T1	Male	101	0.0990	5.27921	104.19	10,523.50	5030.500	−0.407	0.684
	Female	103	−0.5825	5.10579	100.84	10,386.50			
LSS-2/LSS-1 Positive Feedback T1	Male	101	0.3960	4.25929	108.87	10,996.00	4558.000	−1.532	0.125
	Female	103	−0.4951	3.89538	96.25	9914.00			

Note:* *p* < 0.05; M: mean; SD: standard deviation.

**Table 21:** Descriptive statistics and analysis of mean differences considering the LSS-1 and LSS-2 scales at Time 1 based on the course variable for the total sample.

	School Years	n	M	SD	Average Range	Chi-Square	Sig.
LSS-2/LSS-1 Training and Instruction T1	1st SE	31	1.0323	7.40938	110.79	10.469	0.033 *
	2nd SE	45	2.5778	6.57298	122.64		
	3rd SE	42	−0.2143	8.05329	97.13		
	4th SE	55	−1.4545	6.63832	86.15		
	1st HS	31	−0.0645	4.53090	101.26		
	Total	204	0.2794	6.89008			
LSS-2/LSS-1 Democratic Behavior T1	1st SE	31	−0.8387	5.50210	91.90	4.739	0.315
	2nd SE	45	1.6000	6.16220	114.74		
	3rd SE	42	−0.7857	6.00218	92.46		
	4th SE	55	0.2727	6.42176	107.97		
	1st HS	31	−0.2258	4.66697	99.21		
	Total	204	0.1029	5.91518			
LSS-2/LSS-1 Autocratic Behavior T1	1st SE	31	0.2258	3.90478	103.97	16.384	0.003 **
	2nd SE	45	1.9111	3.91281	125.34		
	3rd SE	42	0.7619	2.82678	108.63		
	4th SE	55	−0.1636	4.21972	95.42		
	1st HS	31	−1.0645	2.60686	72.13		
	Total	204	0.4069	3.72532			
LSS-2/LSS-1 Social Support T1	1st SE	31	1.0645	5.15063	116.27	6.830	0.145
	2nd SE	45	0.1556	4.56247	106.58		
	3rd SE	42	0.3333	5.14979	111.92		
	4th SE	55	−1.0364	6.26266	93.99		
	1st HS	31	−1.5161	3.62281	85.15		
	Total	204	−0.2451	5.19081			
LSS-2/LSS-1 Positive Feedback T1	1st SE	31	−0.9032	3.85880	88.35	6.951	0.138
	2nd SE	45	0.9333	3.94058	117.60		
	3rd SE	42	0.3571	4.53077	106.49		
	4th SE	55	−0.0545	4.18736	103.45		
	1st HS	31	−1.1935	3.50637	87.63		
	Total	204	−0.0539	4.09397			

Note: * p < 0.05; ** p < 0.01; M: mean; SD: standard deviation; SE: secondary education; HS: high school.

**Table 22:** Descriptive statistics and analysis of mean differences based on performance level, considering the difference LSS-2 minus LSS-1 for the total student sample at Time 1 (total n = 98).

	Level of Practice	n	M	SD	Average Range	Chi-Square	Sig.
LSS-2/LSS-1 Training and Instruction T1	Leisure	18	1.7222	8.31586	49.25	0.417	0.937
	School Practice	15	0.0667	10.22928	45.50		
	Federated	60	1.1333	6.45576	50.69		
	High Performance	5	0.6000	2.07364	48.10		
LSS-2/LSS-1 Democratic Behavior T1	Leisure	18	−2.3333	7.25177	39.44	3.443	0.328
	School Practice	15	0.5333	5.78010	48.53		
	Federated	60	0.8333	5.77595	51.84		
	High Performance	5	2.4000	5.17687	60.50		
LSS-2/LSS-1 Autocratic Behavior T1	Leisure	18	0.3889	3.05130	43.69	4.669	0.198
	School Practice	15	1.0667	3.49421	49.67		
	Federated	60	1.1167	3.28887	49.12		
	High Performance	5	5.4000	5.68331	74.50		
LSS-2/LSS-1 Social Support T1	Leisure	18	−1.0000	4.33861	43.06	1.662	0.645
	School Practice	15	0.4667	5.95059	54.27		
	Federated	60	0.7000	4.70953	50.71		
	High Performance	5	0.0000	2.12132	43.90		
LSS-2/LSS-1 Positive Feedback T1	Leisure	18	0.0000	4.93487	49.03	0.287	0.962
	School Practice	15	−0.1333	5.13902	53.10		
	Federated	60	0.0000	3.78668	48.79		
	High Performance	5	0.0000	2.54951	48.90		

Note: M: mean; SD: standard deviation.

## 4. Discussion

### 4.1. Importance of Adaptive Leadership in Physical Education

The results obtained confirm the importance of adaptive leadership in the field of physical education. The congruence between perceived and preferred leadership emerges as a crucial factor in increasing student satisfaction. This observation aligns with the postulates of Chelladurai´s [32] Multidimensional Leadership Model, which states that the alignment between the required, perceived, and preferred leadership behaviors enhances group satisfaction.

These findings resonate with recent empirical evidence indicating that autonomy-supportive and transformational leadership behaviors in PE are strongly associated with enhanced student motivation, engagement, competence, and satisfaction. Notably, Behzadnia et al. [33] report that perceived autonomy support correlates positively with student wellness and future physical activity intentions, whereas controlling behaviors link to need frustration and adverse outcomes. Moreover, Fransen et al. [21] underscore that distributed leadership structures—encompassing both task leaders and motivational/social leaders within teams—can significantly reinforce group cohesion and collective efficacy. These recent insights extend our interpretation of the observed preferences for social support and positive feedback, suggesting that leadership in PE should evolve toward more relational, empowering, and shared models, rather than relying solely on traditional autocratic or instructional methods. In this regard, Raiola et al. [6] emphasize that evidence-based and adaptive pedagogical strategies—tailored to the evolving needs of students—are essential for optimizing both immediate learning outcomes and long-term engagement. Similarly, Esposito [10] demonstrates that the competitive context significantly shapes leadership expectations, with more competitive environments fostering stronger preferences for structured yet supportive styles. Ceruso et al. [15] provide complementary evidence from adolescent training programs, showing that emotional reinforcement and personalized feedback not only improve performance but also increase perceived progress and adherence. In line with this, research in competitive sport has shown that the incongruence between preferred and perceived leadership is associated with a higher burnout risk in youth athletes [7], which also reinforces the relevance of achieving congruence in PE contexts to safeguard student well-being.

Collectively, these studies reinforce the present work’s emphasis on adaptive leadership as a driver of long-term student engagement, highlighting that such adaptability should integrate evidence-based pedagogy, sensitivity to competitive contexts, and the deliberate use of emotional reinforcement.

### 4.2. Implications of Gender and Academic Maturity

The preference of female students for democratic leadership styles can be understood through the lens of gender socialization theories and relational leadership models. Research indicates that females often value collaboration, inclusion, and affective support in interpersonal contexts [34,35]. In educational settings, these tendencies translate into a preference for participative and supportive teacher behaviors, which foster a sense of belonging and competence [36]. Esposito [10] observed that female athletes in competitive environments tend to prioritize participatory and supportive leadership approaches. This parallel suggests that gender-sensitive pedagogical adaptations in PE should integrate both collaborative decision-making opportunities and structured emotional support to meet the expectations of female students.

Regarding academic maturity, older students’ greater acceptance of autocratic leadership may reflect their increased orientation toward efficiency and task completion under academic pressure, consistent with developmental theories that suggest a shift from relational to instrumental priorities in late adolescence [36]. However, this interpretation should be viewed cautiously, as the study did not directly measure underlying motives. The patterns observed in this study also align with hierarchical preference structures identified in sport performance contexts [14], where different demographic subgroups systematically rank leadership dimensions according to context-specific priorities. Applying such methodologies in PE research could clarify whether age-related differences stem from developmental changes, accumulated sport experience, or situational demands.

Furthermore, doctoral research on burnout and leadership in youth sport has stressed the formative and educational implications of leadership styles [11], which parallels the gender- and age-related patterns observed in this study and suggests that PE teachers should adopt differentiated strategies according to student characteristics.

The study posits that congruence between perceived and preferred leadership styles enhances student satisfaction; however, since satisfaction was not directly assessed, this conclusion rests on theoretical inference and prior empirical evidence [37]. Specifically, the alignment likely promotes the fulfillment of basic psychological needs—autonomy, competence, and relatedness—which are essential drivers of motivation and engagement. In future research, combining longitudinal tracking with analytical hierarchy methods [14] could provide a more nuanced understanding of how leadership preference patterns evolve and interact with gender and academic maturity.

Future research should include direct measures of satisfaction and engagement to validate these associations and employ longitudinal designs to elucidate causal pathways. This would provide a more robust foundation for pedagogical recommendations aimed at optimizing leadership effectiveness in physical education.

### 4.3. Importance of Social Support and Positive Feedback

Social support and positive feedback were the most-valued factors by students throughout the study. These results underscore the importance of teachers focusing not only on the technical aspects of training but also on the emotional needs of their students. As Weinberg and Gould [2] state, effective sport leaders are those who balance technical demands with emotional support, which not only improves performance but also enhances student satisfaction and overall well-being. Comparable findings in adolescent resistance training programs [15] demonstrate that emotional reinforcement contributes significantly to perceived progress and engagement. In particular, Ceruso and colleagues [15] highlight that constructive feedback, the recognition of effort, and the creation of a supportive climate are central to sustaining adolescents’ motivation—elements that mirror the dimensions most valued by students in the present study.

The preference for more authoritarian leadership styles observed among students in higher academic grades represents an intriguing shift. While younger students tend to favor democratic and supportive behaviors, older students—particularly those in upper secondary education—appear more accepting of autocratic behaviors. This evolution may be explained by developmental psychology theories, which suggest that adolescents nearing adulthood increasingly seek structure, clarity, and efficiency in task-oriented contexts. When such autocratic leadership is coupled with consistent positive feedback and social support, it may be reinterpreted by students not as controlling but as decisive and competence-enhancing, thus maintaining its motivational value. Moreover, school culture might play a significant role; as academic demands intensify in later grades, institutional expectations often prioritize performance and discipline, which may socialize students into valuing directive leadership styles. These factors combined suggest that the preference for authoritarian leadership is not necessarily a rejection of emotional support but, rather, a contextual adaptation to evolving academic and social expectations. However, it is important to note that not all studies fully support this relationship. For instance, some investigations have reported no significant association between leadership behavior and student motivation or have found results influenced by contextual factors such as age, sport type, or cultural differences. This variability reinforces the need for PE leadership models that integrate both structured task-oriented behaviors and relational, emotionally supportive approaches—an integration already advocated for in evidence-based pedagogical frameworks within sport sciences [6]. These contrasting findings highlight the complexity of the topic and suggest that our results should be interpreted within a broader and more nuanced perspective.

### 4.4. Importance of Extracurricular Practice and Practice Level

Student athletes tend to show a greater inclination toward leadership styles that offer more social support and positive feedback, especially when they experience high levels of perfectionism. This suggests that, for these students, effort and commitment in physical education classes may be related to a greater need for emotional and technical support from the teacher [25], especially when facing challenges in reaching their personal performance goals. Evidence from competitive sports [10] indicates that increased competitive involvement heightens expectations for structured and supportive leadership, with a stronger emphasis on clarity of instruction, constructive feedback, and emotional reinforcement. This pattern was also evident in the present study among federated and high-performance athletes, reinforcing the relevance of context-sensitive leadership.

Moreover, students who practice sports seem to perceive their physical education teacher in a manner similar to how they perceive their coaches [38], which could explain a greater congruence between their leadership expectations and the behaviors perceived from the teacher, especially in the factors of training and instruction and democratic behavior. This similarity could be particularly relevant in school contexts where extracurricular sport practice is common. Such parallels are consistent with findings in tennis performance research using the Analytic Hierarchy Process [14], in which competitive athletes demonstrated distinct priority structures in performance factors. This methodological approach could be applied to PE settings to map how varying practice levels shape leadership preference hierarchies, providing a structured tool for tailoring instructional strategies.

These findings converge with previous doctoral evidence [11], which highlighted that young athletes’ experiences of leadership—particularly when linked to burnout risk—are deeply influenced by the competitive environment and the congruence between expected and actual behaviors. Extending this reasoning to PE, extracurricular practice levels appear to shape leadership perceptions in ways comparable to competitive sport.

On the other hand, non-athletic students do not show significant differences in their perceptions of leadership regarding the five factors of the LSS-2 scale, suggesting that, for these students, the teacher’s leadership may be more aligned with the general expectations of the group, without the direct influence of the sports context [39]. This pattern may reflect lower levels of engagement or identification with the PE setting or different motivational profiles not centered on social affiliation or feedback. Drawing on the broader sports science literature, similar dynamics have been observed in individualized rehabilitation and performance programs [16,17,18], where aligning strategies with participant backgrounds and goals significantly improved adherence and perceived progress.

These results reinforce the idea that the extracurricular sports context plays a crucial role in the perception and preference of leadership in physical education, suggesting the need for the teacher to adapt their leadership style according to the characteristics and needs of the students, whether athletic or non-athletic. Such adaptability—integrating evidence-based pedagogy, sensitivity to competitive context, and personalized feedback—could not only improve students’ satisfaction with physical education classes but also optimize their performance and long-term engagement.

### 4.5. Congruence and Satisfaction

The results also reflect that, when physical education teachers align their leadership behaviors with students’ preferences, greater satisfaction is achieved and, consequently, better performance. This congruence between perceived and preferred leadership, especially in the factors of positive feedback and social support, showed the highest degree of congruence and were also most strongly associated with positive student response. These findings are consistent with Self-Determination Theory [37,40,41], which posits that satisfaction of basic psychological needs—autonomy, competence, and relatedness—leads to enhanced intrinsic motivation and commitment. Comparable conclusions can be drawn from individualized strategies in sports medicine—such as tailored rehabilitation for ACL injuries [18], intervention efficacy evaluations [16], and adherence-enhancing modalities like whole-body cryotherapy [17]—all of which show that the alignment between participant needs and program delivery fosters sustained engagement, mirroring the motivational benefits of leadership congruence in PE contexts.

However, a critical insight from this study is that only 36.3% of students exhibited full congruence between perceived and preferred leadership styles across both measurement points. This proportion, while indicative of a positive subgroup, reveals that nearly two-thirds of students experienced some level of mismatch between their expectations and their teachers’ actual behaviors. Such incongruence, as highlighted in competitive sport leadership research [10], may undermine motivation, reduce perceived autonomy, and weaken the classroom climate—especially in contexts where leadership expectations are shaped by prior sport participation.

Such incongruence has also been linked to burnout in sport contexts [7], which underscores the educational relevance of monitoring and adjusting leadership in PE to prevent negative motivational outcomes.

From a pedagogical perspective, this finding underscores the importance of continuous feedback mechanisms and the need for teachers to develop adaptive leadership skills. Incorporating analytical approaches such as the preference hierarchy framework [14] could enable educators to systematically map and track shifts in student priorities across the academic year, providing an evidence-based foundation for instructional adjustments. Initial assessments of students’ leadership preferences at the beginning of the academic year, followed by regular reflection and adjustment, could help close this perceptual gap. Moreover, teacher education programs should incorporate training in relational leadership and inclusive pedagogy to better prepare educators to respond to diverse student expectations. Fostering congruence is not a matter of merely matching styles but of cultivating an educational environment grounded in mutual understanding, responsiveness, and pedagogical flexibility.

### 4.6. Limitations and Future Research

This study involved several limitations that should be considered when interpreting its results. First, the sample consisted exclusively of students from two private schools in Spain, which limits the generalizability of the findings to other educational contexts, particularly public schools or those with different sociocultural profiles. This limitation is particularly relevant because leadership expectations and perceptions are shaped by cultural norms, socioeconomic backgrounds, and the institutional ethos. Consequently, the findings from relatively homogeneous, middle- to upper-middle-class private school contexts may not directly translate to public schools, rural settings, or regions with greater cultural diversity. Comparative studies across varied educational systems and socioeconomic strata are needed to determine the extent to which the patterns observed here are context-specific or generalizable. Future studies should broaden sampling to include diverse educational systems and cultural settings, as recent research in sport sciences [10] has demonstrated that competitive context and institutional culture can substantially shape leadership expectations. Second, although the study adopted a longitudinal design, it included only two measurement points over a one-year interval. A greater number of assessments or a longer follow-up period would have allowed for a deeper analysis of the temporal stability of leadership perceptions and preferences and their potential evolution across different academic stages. Incorporating analytical frameworks such as preference hierarchy analysis [14] could enable researchers to capture subtle shifts in leadership priorities across time and demographic subgroups.

Furthermore, the study focused on a descriptive analysis and non-parametric comparisons, without applying complex inferential models that could provide a deeper understanding of the relationships between leadership, contextual variables, and educational outcomes. Although this decision was aligned with the initial exploratory objective, future research should integrate multivariate statistical approaches, effect size estimation, and confidence intervals, as well as mixed-method designs that combine quantitative and qualitative data. This would allow for triangulation of results and a richer interpretation of the mechanisms underpinning leadership congruence. Additionally, methodological enhancements such as standardized measurement intervals, advanced missing-data handling (e.g., multiple imputation), and the integration of direct measures of satisfaction and engagement would address the current limitations and strengthen causal inference.

Finally, although validated and widely recognized instruments (LSS-1 and LSS-2) were used, the study relied solely on students’ self-perceptions, which may introduce social desirability bias. Future studies should combine objective measures, classroom observations, and teacher interviews to contrast and enrich the information collected. Drawing from evidence on individualized interventions in sports medicine and rehabilitation [16,17,18], such multi-informant and multi-method approaches could ensure that leadership assessments reflect both intended pedagogical strategies and their actual impact on student experience.

Despite these limitations, the findings provide a solid starting point for understanding leadership perceptions and preferences in physical education. Future research may expand the scope by using more representative samples, intervention designs, and more advanced inferential analyses, as well as by formulating concrete pedagogical proposals based on evidence.

The absence of an a priori power analysis and attrition between measurement points may have limited the statistical power for certain subgroup analyses. While missing data were handled using mean substitution within subscales, future research should apply more robust methods such as multiple imputation or maximum likelihood estimation to preserve validity without inflating the Type I or Type II error risk. Furthermore, effect size measures and confidence intervals were not included, which limits the interpretability of the findings. Addressing these methodological gaps will be critical for advancing from exploratory trends toward actionable, evidence-based pedagogical recommendations.

## 5. Conclusions

This longitudinal study provides new evidence on the importance of leadership in physical education. The results emphasize that greater congruence between leadership perceptions and preferences significantly improves student satisfaction and performance. Furthermore, the findings indicate that physical education teachers should adapt their leadership styles not only based on the characteristics of the group but also on individual factors such as gender and academic level. This study contributes to the understanding of how leadership styles in PE should vary by student demographic characteristics. These conclusions are reinforced by recent evidence in sport sciences demonstrating that adaptive, emotionally supportive, and context-sensitive leadership enhances engagement, cohesion, and performance in both educational and athletic settings [6,10,15] and that such approaches align with contemporary pedagogical calls for inclusivity and personalization.

Future studies should explore tertiary and vocational education contexts and diverse sociocultural environments to validate the consistency and developmental trajectory of these findings. Research could also focus on specific interventions to improve teacher leadership in physical education, using experimental or multi-wave longitudinal designs to assess their effects on student outcomes. Contextual variables such as class size, institutional culture, the availability of resources, and socioeconomic background should be systematically addressed, as well as the role of digital tools in teaching practice. For instance, technology-enhanced feedback systems and the real-time monitoring of student perceptions could help align leadership behaviors more closely with evolving preferences. Moreover, incorporating analytic approaches such as those applied in performance priority studies [14] may provide deeper insight into how different student subgroups prioritize leadership dimensions.

Teachers should engage in continuous professional development through targeted leadership workshops, peer-coaching modules, and reflective supervision sessions. It is essential to adapt leadership styles to be inclusive and equitable, considering diversity in gender, academic level, and culture. In line with individualized approaches shown to be effective in sports medicine and training contexts [16,17,18], aligning instructional methods with student needs is likely to foster sustained motivation, participation, and perceived competence in PE. Finally, fostering a positive environment grounded in trust, mutual respect, and ongoing dialogue between teachers and students should be considered a cornerstone of effective leadership in physical education.

## Figures and Tables

**Table 1:** Sociodemographic Data of the Participants.

Variable	Value	
Gender	Female n = 190 (51.35%) Male n = 180 (48.65%) Total n = 370 (100%)	
Age	M = 14.21	SD = 1.41
Academic Level	1st Year of Secondary Education n = 86 (23.31%) 2nd Year of Secondary Education n = 94 (25.47%) 3rd Year of Secondary Education n = 81 (21.95%) 4th Year of Secondary Education n = 77 (20.87%) First year of upper Secondary Education n = 31 (8.40%)	
Sport Practice	Practice n = 302 (82.8%) Not practice n = 63 (17.26%)	
Level of Sport and Physical Activity	High Performance n = 9 (3.98%) Federated n = 127 (56.19%) School Practice n = 49 (21.68%) Leisure n = 41 (18.14%)	

**Table 2:** Descriptive statistics of the prorated LSS-1 scale (from 1 to 5 points) for the total sample at time 1.

	M	SD	Minimum	Maximum
LSS-1 Training and Instruction T1	3.4595	0.56626	2.15	5.00
LSS-1 Democratic Behavior T1	3.1549	0.63133	1.33	5.00
LSS-1 Autocratic Behavior T1	3.2027	0.89635	1.40	5.00
LSS-1 Social Support T1	3.5888	0.69757	1.50	5.00
LSS-1 Positive Feedback T1	3.8326	0.74298	1.40	5.00

M: mean; ST: standard deviation.

**Table 3:** Descriptive statistics of the prorated LSS-2 scale (from 1 to 5 points) for the total sample at time 1.

	M	SD	Minimum	Maximum
LSS-2 Training and Instruction T1	3.4726	0.67029	1.85	5.00
LSS-2 Democratic Behavior T1	3.1651	0.74859	1.22	5.00
LSS-2 Autocratic Behavior T1	3.3048	0.89268	1.40	5.00
LSS-2 Social Support T1	3.5697	0.71376	1.00	5.00
LSS-2 Positive Feedback T1	3.8356	0.76397	1.40	5.00

M: mean; ST: standard deviation.

**Table 4:** Descriptive statistics and mean difference analysis considering the LSS-1 Scale at Time 1 and 2 using the Wilcoxon W test.

	Ranks	n	M	SD	Average Range	Total of Ranks	Z	Sig.
LSS-1 Training and Instruction T1/T2	Negative Ranks	46	46.8991	7.94302	49.96	2298.00	−0.283	0.777
	Positive Ranks	51	47.0917	7.75020	48.14	2455.00		
	Equal Ranks	12						
	Total	109						
LSS-1 Democratic Behavior T1/T2	Negative Ranks	57	28.7248	6.14617	54.75	3121.00	−1.083	0.279
	Positive Ranks	48	27.9266	7.50149	50.92	2444.00		
	Equal Ranks	4						
	Total	109						
LSS-1 Autocratic Behavior T1/T2	Negative Ranks	56	13.7156	3.85403	48.14	2599.50	−1.799	0.072 †
	Positive Ranks	46	13.2752	3.78807	44.17	1678.50		
	Equal Ranks	7						
	Total	109						
LSS-1 Social Support T1/T2	Negative Ranks	56	27.4862	5.83828	52.38	2933.50	−1.026	0.305
	Positive Ranks	46	26.9633	5.98289	50.42	2319.50		
	Equal Ranks	7						
	Total	109						
LSS-1 Positive Feedback T1/T2	Negative Ranks	48	18.6789	3.92966	50.27	2413.00	−0.132	0.895
	Positive Ranks	49	18.6972	4.24256	47.76	2340.00		
	Equal Ranks	12						
	Total	109						

Note: † *p* < 0.10; M: mean; SD: standard deviation.

**Table 5:** Descriptive statistics and analysis of mean differences considering the LSS-2 scale at Time 1 and 2 for the 12-month sample.

	Ranks	n	M	SD	Average Range	Total of Ranks	Z	Sig.
LSS-2 Training and Instruction T1/T2	Negative Ranks	43	48.1724	8.37800	41.69	1792.50	−0.622	0.534
	Positive Ranks	38	47.4598	8.22476	40.22	1528.50		
	Equal Ranks	6						
	Total	87						
LSS-2 Democratic Behavior T1/T2	Negative Ranks	46	28.5057	7.11512	43.00	1978.00	−1.068	0.285
	Positive Ranks	37	27.2989	8.39035	40.76	1508.00		
	Equal Ranks	4						
	Total	87						
LSS-2 Autocratic Behavior T1/T2	Negative Ranks	44	14.2299	4.15037	43.72	1923.50	−1.682	0.092 †
	Positive Ranks	35	13.1379	4.19350	35.33	1236.50		
	Equal Ranks	8						
	Total	87						
LSS-2 Social Support T1/T2	Negative Ranks	43	27.6897	5.84153	43.03	1850.50	−1.108	0.268
	Positive Ranks	37	26.8966	6.84893	37.55	1389.50		
	Equal Ranks	7						
	Total	87						
LSS-2 Positive Feedback T1/T2	Negative Ranks	41	19.1034	3.88807	44.61	1829.00	−1.221	0.222
	Positive Ranks	38	18.6322	3.76061	35.03	1331.00		
	Equal Ranks	8						
	Total	87						

Note: † *p* < 0.10; M: mean; SD: standard deviation.

**Table 6:** Descriptive statistics and analysis of mean differences based on the gender/sex variable considering the LSS-1 scale and Time 1 (total n = 211).

	Gender	n	M	SD	Average Range	Total of Ranks	Mann–Whitney U	Z	Sig.
LSS-1 training and instruction T1	Male	112	46.0804	7.63012	121.77	13,638.50	4897.500	−2.542	0.011 *
	Female	109	43.8349	6.92622	99.93	10,892.50			
LSS-1 Democratic Behavior T1	Male	112	29.0714	5.87668	118.72	13,297.00	5239.000	−1.824	0.068 †
	Female	109	27.6972	5.41347	103.06	11,234.00			
LSS-1 Autocratic Behavior T1	Male	112	16.0536	4.40074	111.62	12,501.00	6035.000	−0.146	0.884
	Female	109	15.9725	4.58350	110.37	12,030.00			
LSS-1 Social Support T1	Male	112	29.1429	5.66299	117.50	13,159.50	5376.500	−1.534	0.017 *
	Female	109	28.2661	5.48507	104.33	11,371.50			
LSS-1 Positive Feedback T1	Male	112	19.5000	3.96380	118.54	13,277.00	5259.000	−1.784	0.074†
	Female	109	18.8165	3.42422	103.25	11,254.00			

Note: † *p* < 0.10; * *p* < 0.05; M: mean; SD: standard deviation.

**Table 7:** Descriptive statistics and analysis of mean differences considering the LSS-2 scale at Time 1 (total n = 208).

	Gender	n	M	SD	Average Range	Total of Ranks	Mann–Whitney U	Z	Sig.
LSS-2 Training and Instruction T1	Male	102	46.8824	9.41485	116.86	11,920.00	4145.000	−2.909	0.004 **
	Female	106	43.4717	7.65967	92.60	9816.00			
LSS-2 Democratic Behavior T1	Male	102	30.0392	7.01190	117.78	12,013.50	4051.500	−3.126	0.002 **
	Female	106	26.9906	6.13110	91.72	9722.50			
LSS-2 Autocratic Behavior T1	Male	102	16.3824	4.53380	101.89	10,393.00	5140.000	−2.627	0.539
	Female	106	16.6604	4.41186	107.01	10,343.00			
LSS-2 Social Support T1	Male	102	29.1961	5.98023	111.39	11,361.50	4703.500	−1.622	0.105
	Female	106	27.9434	5.39458	97.87	10,374.50			
LSS-2 Positive Feedback T1	Male	102	19.8333	3.88213	116.09	11,841.50	4223.500	−2.736	0.006 **
	Female	106	18.5472	3.66744	93.34	9894.50			

Note: ** *p* < 0.01; M: mean; SD: standard deviation.

**Table 8:** Descriptive statistics and analysis of mean differences considering the LSS-1 scale and the school year based on the general sample and applying the Kruskal-Wallis test for mean differences.

	School Year	n	M	SD	Average Range	Chi-Square	Sig.
LSS-1 Training and Instruction T1	1 SE	39	48.0000	7.98309	136.93	36.176	0.000 ***
	2 SE	51	47.4118	7.19771	132.02		
	3 SE	46	46.5652	7.96842	126.25		
	4 SE	55	41.1273	5.26010	77.62		
	1 HS	20	41.7097	4.86617	81.23		
	Total	221	44.9729	7.36140			
LSS-1 Democratic Behavior T1	1 SE	39	29.9211	6.44050	127.92	8.783	0.067 †
	2 SE	51	29.1569	5.99124	118.18		
	3 SE	46	28.1087	6.32536	108.14		
	4 SE	55	26.6909	4.76046	91.59		
	1 HS	20	28.7097	3.93441	117.13		
	Total	221	28.3937	5.68201			
LSS-1 Autocratic Behavior T1	1 SE	39	14.8158	4.49537	93.37	86.100	0.000 ***
	2 SE	51	14.3529	3.67600	87.26		
	3 SE	46	12.5652	3.01590	61.97		
	4 SE	55	19.3091	3.63049	157.65		
	1 HS	20	19.4839	2.32194	161.66		
	Total	221	16.0136	4.48176			
LSS-1 Social Support T1	1 SE	39	28.5526	5.22312	107.14	13.431	0.009 **
	2 SE	51	28.5882	4.94440	106.37		
	3 SE	46	26.1522	6.40821	86.80		
	4 SE	55	30.2182	5.79492	129.30		
	1 HS	20	30.2258	3.91331	126.77		
	Total	221	28.7104	5.58059			
LSS-1 Positive Feedback T1	1 SE	39	19.6053	3.43700	118.72	6.267	0.180
	2 SE	51	18.6863	3.33761	99.89		
	3 SE	46	18.2826	4.58821	99.12		
	4 SE	55	19.4364	3.58267	116.41		
	1 HS	20	20.2258	2.57824	127.84		
	Total	221	19.1629	3.71492			

Note: † *p* < 0.10; ** *p* < 0.01; *** p < 0.001; M: mean; SD: standard deviation; SE: secondary education; HS; high school.

**Table 9:** Descriptive statistics and mean difference analysis considering the LSS-2 Scale and the course variable at Time 1.

	School Year	n	M	SD	Average Range	Chi-Square	Sig.
LSS-2 Training and Instruction T1	1st SE	32	48.8750	8.51090	133.42	54.549	*p* < 0.001
	2nd SE	46	50.1087	7.92248	139.48		
	3rd SE	43	46.7907	8.81708	115.34		
	4th SE	56	39.6071	6.22511	64.04		
	1st HS	31	41.6452.	6.97399	80.81		
	Total	208	45.1442	8.71383			
LSS-2 Democratic Behavior T1	1st SE	32	28.6563	7.88469	107.41	9.547	0.049 *
	2nd SE	46	31.2391	6.90953	126.18		
	3rd SE	43	27.3721	7.50245	94.42		
	4th SE	56	26.9821	5.42215	92.40		
	1st HS	31	28.4839	5.19532	105.16		
	Total	208	28.4856	6.73729			
LSS-2 Autocratic Behavior T1	1st SE	32	14.6875	4.33618	80.16	54.231	*p* < 0.001
	2nd SE	46	16.1957	4.38492	98.91		
	3rd SE	43	13.3721	3.70343	61.78		
	4th SE	56	19.2143	3.82201	140.95		
	1st HS	31	18.4194	2.82576	131.34		
	Total	208	16.5240	4.46342			
LSS-2 Social Support T1	1st SE	32	29.2813	6.26426	113.80	6.837	0.145
	2nd SE	46	29.0652	4.80001	109.09		
	3rd SE	43	26.5116	6.63459	83.92		
	4th SE	56	29.2143	5.53900	111.47		
	1st HS	31	28.7097	4.92066	104.05		
	Total	208	28.5577	5.71011			
LSS-2 Positive Feedback T1	1st SE	32	18.6563	4.40388	98.94	2.125	0.713
	2nd SE	46	19.7826	3.72924	114.34		
	3rd SE	43	18.6512	4.18584	97.64		
	4th SE	56	19.4643	3.23596	105.77		
	1st HS	31	19.0323	3.82521	102.87		
	Total	208	19.1779	3.81987			

Note: * p < 0.05; M: mean; SD: standard deviation; SE: secondary education; HS: high school.

**Table 10:** Descriptive statistics and mean difference analysis considering the LSS-1 scale and the course based on the male sample using the Kruskal–Wallis test.

	School Year	n	M	SD	Average Range	Chi-Square	Sig.
LSS-1 Training and Instruction T1	1st SE	32	49.6667	7.25119	71.48	26.539	*p* < 0.001
	2nd SE	46	49.3929	7.19228	71.34		
	3rd SE	43	45.8261	7.45686	56.57		
	4th SE	56	41.1000	5.83005	34.84		
	1st HS	31	41.7647	5.90052	36.97		
	Total	208	46.0804	7.63012			
LSS-1 Democratic Behavior T1	1st SE	32	30.9167	6.49359	69.38	8.632	0.071 †
	2nd SE	46	29.7500	5.89177	58.86		
	3rd SE	43	29.0435	6.51207	54.52		
	4th SE	56	26.1000	5.26058	40.98		
	1st HS	31	28.8824	3.49790	55.38		
	Total	208	29.0714	5.87668			
LSS-1 Autocratic Behavior T1	1st SE	32	15.7917	4.27306	55.02	33.001	*p* < 0.001
	2nd SE	46	15.3214	3.72234	50.55		
	3rd SE	43	12.5217	3.32852	30.59		
	4th SE	56	18.5000	4.67355	73.43		
	1st HS	31	19.5294	2.06152	83.53		
	Total	208	16.0536	4.40074			
LSS-1 Social Support T1	1st SE	32	29.7500	4.87451	60.27	4.799	0.309
	2nd SE	46	29.7857	5.20937	58.39		
	3rd SE	43	26.5652	6.46548	43.59		
	4th SE	56	29.8000	7.22277	62.35		
	1st HS	31	29.9412	3.32548	58.65		
	Total	208	29.1429	5.66299			
LSS-1 Positive Feedback T1	1st SE	32	20.2500	3.50466	62.25	1.987	0.738
	2nd SE	46	19.2500	3.39526	52.02		
	3rd SE	43	19.1739	4.75444	56.26		
	4th SE	56	18.6500	5.15318	52.25		
	1st HS	31	20.2941	2.56819	61.09		
	Total	208	19.5000	3.96380			

Note: † *p* < 0.10; M: mean; SD: standard deviation; SE: secondary education; HS: high school.

**Table 11:** Descriptive statistics and mean difference analysis considering the LSS-1 Scale and the course based on the female sample using the Kruskal–Wallis test.

	School Year	n	M	SD	Average Range	Chi-Square	Sig.
LSS-1 Training and Instruction T1	1st SE	14	45.1429	8.62784	59.36	10.381	0.034 *
	2nd SE	23	45.0000	6.57129	60.09		
	3rd SE	23	47.3043	8.51563	68.87		
	4th SE	35	41.1429	4.99496	44.54		
	1st HS	14	41.6429	3.43303	45.64		
	Total	109	43.8349	6.92622			
LSS-1 Democratic Behavior T1	1st SE	14	28.2143	6.20395	53.57	1.648	0.800
	2nd SE	23	28.4348	6.16313	59.50		
	3rd SE	23	27.1739	6.13227	53.78		
	4th SE	35	27.0286	4.49500	50.94		
	1st HS	14	28.5000	4.53618	61.18		
	Total	109	27.6972	5.41347			
LSS-1 Autocratic Behavior T1	1st SE	14	13.1429	4.52101	34.89	58.576	*p* < 0.001
	2nd SE	23	13.1739	3.32555	36.02		
	3rd SE	23	12.6087	2.74258	31.85		
	4th SE	35	19.7714	2.85003	81.13		
	1st HS	14	19.4286	2.68082	79.00		
	Total	109	15.9725	4.58350			
LSS-1 Social Support T1	1st SE	14	26.5000	5.33133	40.14	15.235	0.004 **
	2nd SE	23	27.1304	4.26726	47.22		
	3rd SE	23	25.7391	6.46823	44.43		
	4th SE	35	30.4571	4.90104	67.80		
	1st HS	14	30.5714	4.63622	68.00		
	Total	109	28.2661	5.48507			
LSS-1 Positive Feedback T1	1st SE	14	18.5000	3.13172	52.54	10.530	0.032 *
	2nd SE	23	18.0000	3.29511	46.76		
	3rd SE	23	17.3913	4.33526	42.67		
	4th SE	35	19.8857	2.85710	64.77		
	1st HS	14	20.1429	2.68492	66.82		
	Total	109	18.8165	3.42422			

Note: * *p* < 0.05; ** *p* < 0.01; M: mean; SD: standard deviation; SE: secondary education; HS: high school.

**Table 12:** Descriptive statistics and mean difference analysis considering the LSS-2 Scale and the course variable at Time 1 for the male sample.

	School Year	n	M	SD	Average Range	Chi-Square	Sig.
LSS-2 Training and Instruction T1	1st SE	20	50.5500	9.04652	65.23	30.402	*p* < 0.001
	2nd SE	25	52.4800	7.55050	69.52		
	3rd SE	20	47.2000	9.04724	52.10		
	4th SE	20	40.9000	7.15909	31.70		
	1st HS	17	41.0000	8.44837	31.44		
	Total	102	46.8824	9.41485			
LSS-2 Democratic Behavior T1	1st SE	20	30.5500	7.86381	55.43	8.021	0.091 †
	2nd SE	25	33.3200	6.63777	63.96		
	3rd SE	20	29.2500	6.85085	46.83		
	4th SE	20	27.6500	6.76115	43.60		
	1st HS	17	28.3529	5.72212	43.35		
	Total	102	30.0392	7.01190			
LSS-2 Autocratic Behavior T1	1st SE	20	14.9000	4.39976	41.80	23.212	*p* < 0.001
	2nd SE	25	17.0800	4.44335	54.96		
	3rd SE	20	12.9500	3.77631	29.33		
	4th SE	20	18.7500	4.36342	66.78		
	1st HS	17	18.3529	2.87100	65.94		
	Total	102	16.3824	4.53380			
LSS-2 Social Support T1	1st SE	20	30.4500	6.60522	60.88	4.938	0.294
	2nd SE	25	30.2000	4.39697	55.06		
	3rd SE	20	27.5500	6.13424	41.98		
	4th SE	20	28.9000	7.06288	51.40		
	1st HS	17	28.5494	5.78919	46.56		
	Total	102	29.1961	5.98023			
LSS-2 Positive Feedback T1	1st SE	20	20.0000	4.12948	53.60	3.534	0.473
	2nd SE	25	20.6000	3.74166	58.28		
	3rd SE	20	20.1500	3.80132	53.55		
	4th SE	20	19.1500	3.88350	45.38		
	1st HS	17	18.9412	4.03842	43.85		
	Total	102	19.8333	3.88213			

Note: † *p* < 0.10; M: mean; SD: standard deviation; SE: secondary education; HS: high school.

**Table 13:** Descriptive statistics and mean difference analysis considering the LSS-2 Scale and the course variable at Time 1 for the female sample.

	School Year	n	M	SD	Average Range	Chi-Square	Sig.
LSS-2 Training and Instruction T1	1st SE	12	46.0833	7.01243	45.29	24.307	*p* < 0.001
	2nd SE	21	47.2857	7.57722	61.79		
	3rd SE	23	46.4348	8.79993	47.46		
	4th SE	36	38.8889	5.62026	51.53		
	1st HS	14	42.4286	4.81527	63.11		
	Total	106	43.4717	7.65967			
LSS-2 Democratic Behavior T1	1st SE	12	25.5000	7.14143	38.00	4.806	0.308
	2nd SE	21	28.7619	6.53379	43.19		
	3rd SE	23	25.7391	7.80569	32.76		
	4th SE	36	26.6111	4.58119	73.01		
	1st HS	14	28.6429	4.68397	66.14		
	Total	106	29.9906	6.13110			
LSS-2 Autocratic Behavior T1	1st SE	12	14.3333	4.39697	46.42	32.913	*p* < 0.001
	2nd SE	21	15.1429	4.17475	52.67		
	3rd SE	23	13.7391	3.68310	42.65		
	4th SE	36	19.4722	3.52531	61.36		
	1st HS	14	18.5000	2.87563	58.43		
	Total	106	16.6604	4.41186			
LSS-2 Social Support T1	1st SE	12	27.3333	5.34846	35.42	6.254	0.181
	2nd SE	21	27.7143	5.01142	54.48		
	3rd SE	23	25.6087	7.05007	46.04		
	4th SE	36	29.3889	4.58742	61.25		
	1st HS	14	28.9286	3.81221	59.86		
	Total	106	27.9434	5.39458			
LSS-2 Positive Feedback T1	1st SE	12	16.4167	4.05549	45.29	8.488	0.075 †
	2nd SE	21	18.8095	3.55836	61.79		
	3rd SE	23	17.3478	4.14080	47.46		
	4th SE	36	19.6389	2.86010	51.53		
	1st HS	14	19.1429	3.69734	49.46		
	Total	106	18.5472	3.66744			

Note: † *p* < 0.10; M: mean; SD: standard deviation; SE: secondary education; HS: high school.

**Table 14:** Descriptive statistics and analysis of mean differences between students who practice and do not practice extracurricular sports, considering the Leadership Preferences Questionnaire LSS-1 using the Mann–Whitney U test statistic (total n = 220).

	Sport	n	M	SD	Average Range	Total of Ranks	Mann–Whitney U	Z	Sig.
LSS-1 Training and Instruction T1	Practice	187	44.8717	7.17407	109.81	20,534.00	2956.000	−0.385	0.701
	Not practice	33	45.7273	8.46416	114.42	3776.00			
LSS-1 Democratic Behavior T1	Practice	187	28.4064	5.44134	111.24	20,802.00	2947.000	−0.412	0.681
	Not practice	33	28.6061	6.85994	106.30	3508.00			
LSS-1 Autocratic Behavior T1	Practice	187	16.0802	4.47201	111.35	20,822.00	2927.000	−0.471	0.637
	Not practice	33	15.7879	4.58092	105.70	3488.00			
LSS-1 Social Support T1	Practice	187	29.0107	5.51244	114.34	21,381.50	2367.500	−2.134	0.033 *
	Not practice	33	27.0606	5.83598	88.74	2928.50			
LSS-1 Positive Feedback T1	Practice	187	19.3048	3.73420	113.25	21,177.00	2572.000	−1.528	0.126
	Not practice	33	18.4848	3.56301	94.94	3133.00			

Note: * *p* < 0.05; M: mean; SD: standard deviation.

**Table 15:** Descriptive statistics and analysis of mean differences based on performance level, considering the LSS-1 questionnaire for the total student sample at Time 1.

	Level of Practice	n	M	SD	Average Range	Chi-Square	Sig.
LSS-1 training and instruction T1	Leisure	17	43.1765	8.07957	34.09	9.347	0.025 *
	School Practice	14	45.7857	6.68153	42.04		
	Federated	63	48.2540	7.41613	54.98		
	High Performance	5	50.8000	6.37966	63.70		
	Total	99	47.1616	7.58315			
LSS-1 Democratic Behavior T1	Leisure	17	27.5294	6.42256	41.76	2.605	0.457
	School Practice	14	28.6429	4.68397	46.18		
	Federated	63	29.7143	5.60653	52.33		
	High Performance	5	30.8000	5.06952	59.40		
	Total	99	29.2424	5.61174			
LSS-1 Autocratic Behavior T1	Leisure	17	13.0000	2.89396	47.21	3.536	0.316
	School Practice	14	12.6429	2.34052	41.14		
	Federated	63	14.1587	3.68172	53.71		
	High Performance	5	11.6000	3.36155	37.50		
	Total	99	13.6162	3.42472			
LSS-1 Social Support T1	Leisure	17	26.4706	5.19757	41.59	3.024	0.388
	School Practice	14	27.5000	4.25622	45.57		
	Federated	63	28.3968	5.64983	52.34		
	High Performance	5	30.2000	3.42053	61.50		
	Total	99	28.0303	5.31738			
LSS-1 Positive Feedback T1	Leisure	17	17.6471	4.28575	39.91	8.177	0.042 *
	School Practice	14	17.4286	3.65249	36.68		
	Federated	63	19.6825	3.60477	54.43		
	High Performance	5	21.2000	2.48998	65.80		
	Total	99	19.0909	3.80143			

Note: * *p* < 0.05; M: mean; SD: standard deviation.

**Table 16:** Descriptive statistics and mean difference analysis between students who practice and do not practice extracurricular sports using the LSS-2 Leadership Preferences Questionnaire and the Mann–Whitney U test (total n = 207).

	Sport Practice	n	M	SD	Average Range	Total of Ranks	Mann–Whitney U	Z	Sig.
LSS-2 Training and Instruction T1	Practice	179	44.8603	8.61130	101.92	18.243.00	2133.000	−1.267	0.205
	Not Practice	28	46.9643	9.44960	117.32	3285.00			
LSS-2 Democratic Behavior T1	Practice	179	28.4190	6.42921	102.85	18,410.50	2300.500	−0.698	0.485
	Not Practice	28	29.1071	8.59948	111.34	3117.50			
LSS-2 Autocratic Behavior T1	Practice	179	28.6201	5.54155	103.33	18,496.50	2386.500	−0.406	0.684
	Not Practice	28	28.4286	6.73536	108.27	3031.50			
LSS-2 Social Support T1	Practice	179	28.6201	5.54155	103.36	18,502.00	2392.000	−0.387	0.698
	Not Practice	28	28.4286	6.73536	108.07	3026.00			
LSS-2 Positive Feedback T1	Practice	179	19.2514	3.70673	104.54	18,712.50	2409.500	−0.329	0.742
	Not Practice	28	18.7143	4.58546	100.55	2815.50			

Note: M: mean; SD: standard deviation.

**Table 17:** Descriptive statistics and mean difference analysis based on performance level using the LSS-2 Questionnaire for the Total Student Sample at Time 1.

	Level of Practice	n	M	SD	Average Range	Chi-Square	Sig.
LSS-2 Training and Instruction T1	Leisure	19	46.2105	9.36648	41.16	3.790	0.150
	School Practice	16	46.5625	8.10735	41.25		
	Federated	61	49.9508	7.46643	52.69		
	High Performance	5	51.4000	5.17687	33.66		
	Total	101	48.7822	7.96191	47.69		
LSS-2 Democratic Behavior T1	Leisure	19	25.7368	5.16228	53.34	7.265	0.026 *
	School Practice	16	29.2500	5.70964	41.87		
	Federated	61	30.4098	7.81105	44.69		
	High Performance	5	33.2000	1.92354	51.57		
	Total	101	29.4851	7.09452	38.16		
LSS-2 Autocratic Behavior T1	Leisure	19	13.6316	3.21819	46.31	2.128	0.345
	School Practice	16	14.0625	3.54906	52.30		
	Federated	61	15.1803	4.57350	44.18		
	High Performance	5	17.0000	3.80789	34.00		
	Total	101	14.8020	4.19051	53.65		
LSS-2 Autocratic Behavior T1	Leisure	19	26.1053	5.71445	41.16	3.865	0.145
	School Practice	16	28.0625	4.76751	41.25		
	Federated	61	29.1148	5.81119	52.69		
	High Performance	5	30.2000	2.77489	33.66		
	Total	101	28.4356	5.59896	47.69		
LSS-2 Positive Feedback T1	Leisure	19	18.3158	4.55891	53.34	6.920	0.031 *
	School Practice	16	17.2500	3.02214	41.87		
	Federated	61	19.7541	4.15795	44.69		
	High Performance	5	21.2000	0.83666	51.57		
	Total	101	19.1584	4.08101	38.16		

Note: * *p* < 0.05; M: mean; SD: standard deviation.

## Data Availability

The data presented in this study are available on request from the corresponding author due to privacy restrictions.

## Abbreviations

The following abbreviations are used in this manuscript:

LSS	Leadership Scale for Sport
LSS-1	Leadership Preferences Scale for Sport
LSS-2	Leadership Perceptions Scale for Sport
